# BRCA mutation and multiple primary malignancies: a rare case of recurring triple-negative breast cancer and cervical cancer

**DOI:** 10.3332/ecancer.2025.1939

**Published:** 2025-07-02

**Authors:** Meryem Naciri, Fatima Ezzahra Aouzah, Adil El Ghanmi, Bouchra Ghazi, Karima Fichtali, Sqalli Houssaini Mohammed, Fadila Kouhen

**Affiliations:** 1 Mohammed VI Faculty of Medicine, Mohammed VI University of Sciences and Health (UM6SS), Rabat 10100, Morocco; 2 Mohammed VI Faculty of Medicine, Mohammed VI University of Sciences and Health (UM6SS), Casablanca 20230, Morocco; 3 Department of Radiotherapy, International University Hospital Cheikh Khalifa, Casablanca 20230, Morocco; 4 Department of Gynecology and Obstetrics, Mohammed VI International University Hospital, Bouskoura 20230, Morocco; 5 Laboratory of Neurooncology, Oncogenetic and Personalized Medicine, Faculty of Medicine, Mohammed VI University of Sciences and Health (UM6SS), Casablanca 20230, Morocco; 6 Department of Medical Oncology, International University Hospital Cheikh Khalifa, Casablanca 20230, Morocco; 7 Immunopathology-Immunotherapy-Immunomonitoring Laboratory, Faculty of Medicine, Mohammed VI University of Sciences and Health (UM6SS), Casablanca 20230, Morocco

**Keywords:** BRCA- breast cancer- multiple primary malignancies

## Abstract

Mutations in the BRCA1 and BRCA2 genes significantly increase the risk of hereditary cancers, mainly of the breast and ovary, but also of other cancers such as those of the pancreas, prostate and cervix. In carriers of these mutations, multiple primary malignancies (MPM) represent a complex clinical challenge, influenced by genetic and environmental factors, as well as previous cancer treatments. The case reports a patient with a BRCA1 mutation with a family history of breast and ovarian cancer and who developed cervical cancer then recurrent triple-negative breast cancer treated with mastectomy, radiotherapy, chemotherapy and Poly (Adenosine diphosohate-ribose) polymérase inhibitors. This case underlines the interplay between different malignancies in the context of breast cancer mutations and the importance of specific and personalised treatment of patients with multiple primary malignancies.

## Introduction

BRCA1 and BRCA2 mutations are widely recognised as significant risk factors for hereditary breast and ovarian cancers, accounting for a substantial proportion of these malignancies [1]. These mutations impair the DNA repair mechanisms governed by homologous recombination, leading to genomic instability and an increased predisposition to cancer [2]. While breast and ovarian cancers are the hallmark malignancies associated with breast cancer (BRCA) mutations, emerging evidence suggests that carriers of BRCA1 and BRCA2 mutations may also face an elevated risk for other cancers, including pancreatic, prostate and, as in some reported cases, cervical cancer [1,3].

Multiple primary malignancies (MPMs) are a rare but clinically significant phenomenon, defined as the occurrence of two or more distinct malignancies within the same individual, either synchronously or metachronously [4]. These cases present unique challenges in diagnosis, treatment and long-term management. In patients with BRCA mutations, the occurrence of MPMs may not only reflect the inherited genetic susceptibility but also be influenced by environmental, hormonal and lifestyle factors, as well as the potential cumulative effects of previous cancer treatments such as chemotherapy and radiation [4].

This case reports a patient with a BRCA1 mutation who developed multiple malignancies, including cervical cancer and recurrent triple-negative breast cancer (TNBC). It underscores the wider implications of BRCA mutations, emphasising their association with diverse cancers and the need for ongoing research into targeted therapies for hereditary cancer syndromes.

## Case report

A 46-year-old Moroccan woman, with a family history of ovarian cancer in her mother and bilateral breast cancer in her maternal aunt, presents a medical history of multiple malignancies.

**Figure 1.**
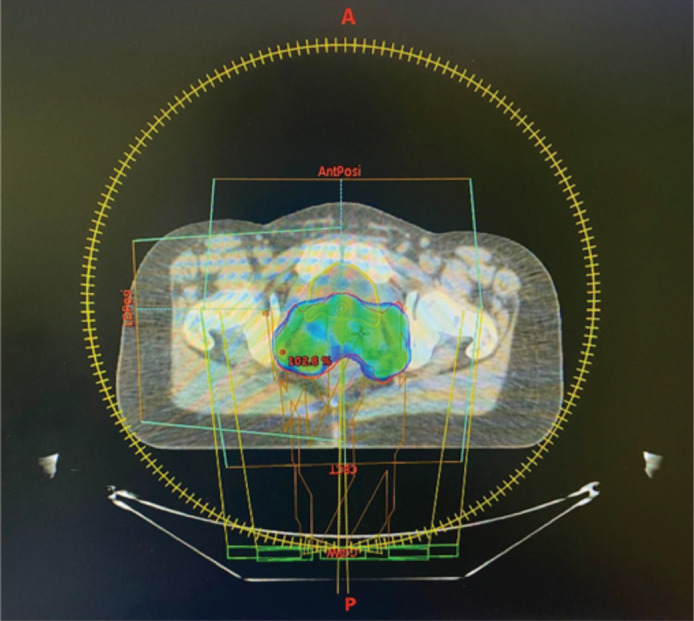
Dosimetric planning for cervical cancer radiotherapy.

At the age of 30, she was diagnosed with FIGO stage IIB squamous cell carcinoma of the cervix. She had never undergone human papillomavirus (HPV) vaccination or cervical cancer screening before her diagnosis. HPV testing at the time confirmed HPV positivity. She was treated with concurrent chemoradiation and brachytherapy (Figure 1), achieving complete remission confirmed by follow-up pelvic Magnetic resonance imaging (MRI) scans. Given the high prevalence of HPV-related cervical cancer in certain regions, the interplay between her HPV status and BRCA mutation warrants further discussion.

At age 36, she was diagnosed with TNBC in the left breast, characterised as an invasive ductal carcinoma of no special type, Scarff, Bloom and Richardson (SBR) Grade III, with a Ki-67 proliferative index of 60%. Immunohistochemistry confirmed the absence of estrogen receptor, progesterone receptor and Human Epidermal Growth Factor Receptor-2 (HER2) expression. The tumour was staged as T2N1M0, with axillary lymph node involvement. She underwent a left mastectomy with axillary lymph node dissection, followed by adjuvant chemotherapy, including platinum-based regimens plus external beam radiotherapy. She remained in remission for several years.

**Figure 2.**
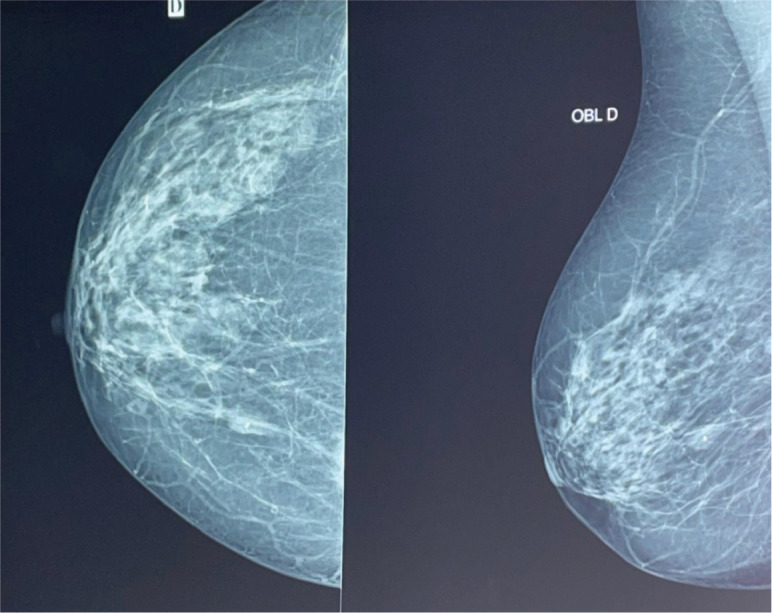
Mammogram showing breast cancer.

**Figure 3.**
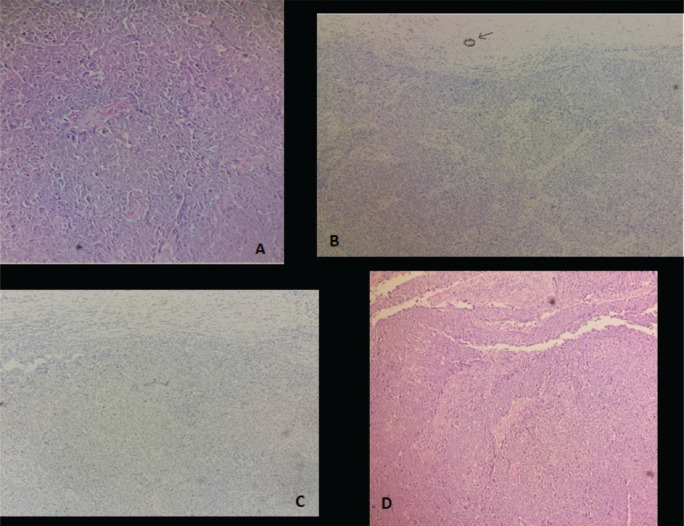
Representative micrograph of the breast tumour (a: Tumour cells present large nuclei, marked variation and prominent nucleoli, b: The tumour cells are negative for estrogen receptors, c: The tumour cells are negative for HER2 and d: Lesion shows high-grade invasive breast cancer of no special type).

At age 45, she identified a lump in her right breast during self-examination. Imaging confirmed a 21 × 15 mm nodule in the upper inner quadrant of the right breast, classified as BIRADS 4 (Figure 2). A biopsy revealed invasive ductal carcinoma, SBR Grade III, negative for hormone receptors and HER2, with a Ki-67 proliferative index of 50% (Figure 3).

Staging included a brain MRI, which was normal, and bone scintigraphy, which identified small hyperfixing foci considered likely benign but requiring follow-up. An ^18^F-FDG PET scan showed hypermetabolism in the right breast (SUV 5.96) and a right axillary lymph node (SUV 2.02), confirming locoregional spreading disease without distant metastases. The cancer was staged as T2N1M0.

Neoadjuvant chemotherapy with carboplatin and paclitaxel was initiated. After three cycles, follow-up imaging showed tumour shrinkage to 13 × 6 mm, prompting an additional three cycles with a weekly carboplatin protocol due to treatment tolerance issues. Surgery followed, with a right mastectomy and axillary lymph node dissection. Pathology revealed a 1 cm residual tumour, SBR Grade II, with no lymph node involvement. The cancer was reclassified as ypT1bN0Mx.

**Figure 4.**
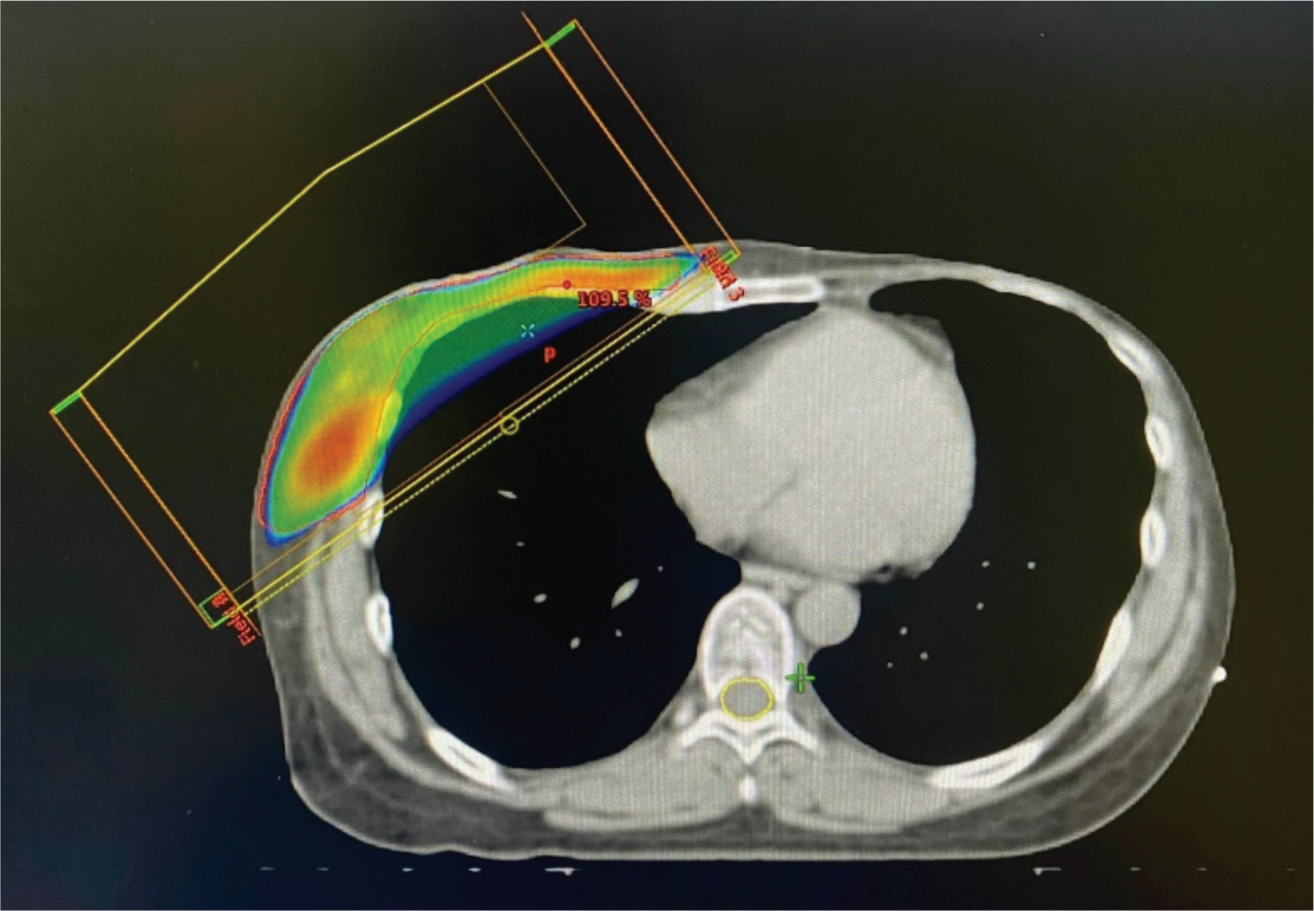
Dosimetric planning for breast cancer radiotherapy.

Adjuvant therapy included capecitabine chemotherapy and radiotherapy to the chest wall and supraclavicular and infraclavicular lymph nodes, delivered at 42 Gray using a 3D conformal field-in-field technique (Figure 4). Genetic consultation confirmed the presence of a BRCA mutation in the patient. Notably, genetic testing of all female family members revealed no mutations, suggesting either a de novo mutation or a non-penetrant family history.

Given her BRCA mutation and history of TNBC, adjuvant olaparib (300 mg twice daily) was included in her treatment plan. The patient is currently under close surveillance, with regular imaging, clinical evaluations and laboratory tests to monitor for recurrence and assess treatment efficacy. Surveillance also includes monitoring for any side effects related to olaparib, ensuring early intervention if needed.

## Discussion

The development of MPMs in patients with BRCA mutations is rare but increasingly recognised, and this case provides an intriguing example of the complex relationship between genetic mutations and cancer susceptibility. BRCA1 mutations are classically linked to an increased risk of breast and ovarian cancers, but they have also been associated with higher risks for other malignancies, including pancreatic, prostate and, as seen in this case, cervical cancer [1]. Understanding how BRCA mutations influence cancer development in a broader context is essential for guiding clinical management, treatment strategies and surveillance protocols.

One of the most striking aspects of this case is the recurrence of TNBC in a patient with a BRCA1 mutation. TNBC is known for its aggressive clinical course, high risk of recurrence and poor prognosis, particularly when compared to other subtypes of breast cancer [5]. Studies have shown that women with BRCA mutations, particularly BRCA1 mutations, are at a higher risk of developing TNBC, which is often characterised by its lack of hormone receptor (estrogen and progesterone) and HER2 expression [6]. This subtype tends to be more challenging to treat because it does not respond to traditional hormonal therapies or HER2-targeted therapies, which are standard treatments for other breast cancer subtypes. In the case presented, despite aggressive treatment modalities, the patient experienced a recurrence of TNBC. This highlights the heightened risk of recurrence in BRCA mutation carriers, especially when dealing with TNBC, which is already an inherently aggressive form of breast cancer.

In addition to the recurring TNBC, the development of cervical cancer in this patient is particularly noteworthy. Cervical cancer is most commonly associated with persistent infection with high-risk strains of HPV, and HPV vaccination has been shown to significantly reduce the incidence of cervical cancers [7]. However, the patient’s BRCA1 mutation could have further predisposed her to developing cervical cancer, a phenomenon observed in some studies that suggest women with BRCA mutations may have a higher risk of HPV-related malignancies [8]. The BRCA1 gene plays a crucial role in the repair of double-strand DNA breaks, and mutations in this gene lead to genomic instability, which may increase susceptibility to carcinogenic processes, including those driven by HPV [9]. The immune system’s ability to clear HPV infections could be compromised in individuals with defective DNA repair mechanisms, allowing the virus to persist and contribute to the development of cervical cancer [10]. Although the majority of cervical cancers are HPV-driven, this case underscores the potential synergistic effect of a BRCA1 mutation in facilitating the development of an HPV-associated malignancy. It is particularly notable that our patient developed cervical cancer at the age of 30, which is highly unusual, as cervical cancer typically occurs in older women, often after the age of 40. This early onset of cervical cancer in the context of a BRCA1 mutation may point to an increased risk in younger individuals with genetic predispositions.

Furthermore, the family history of cancer in this patient, a mother with ovarian cancer and a maternal aunt with breast cancer, strengthens the hypothesis that the BRCA1 mutation played a central role in the development of these multiple cancers. A strong family history of cancers, especially those in close relatives, is a major indicator of the potential presence of a hereditary cancer syndrome, and in this case, it prompted genetic testing [11]. Identification of the BRCA1 mutation allowed the healthcare team to adopt a more aggressive and proactive approach to monitoring for other potential cancers. Genetic counseling in such cases is critical, not only for the patient but also for their family members, who may be at risk and could benefit from genetic testing and early screening.

The management of this patient highlights the importance of personalised care for individuals with BRCA mutations, especially when multiple primary malignancies are involved. Standard treatment regimens for TNBC—typically including chemotherapy with agents such as paclitaxel, anthracyclines and platinum-based drugs—may need to be adjusted based on the patient’s genetic makeup and the nature of the malignancies. In this case, the use of platinum-based chemotherapy, which is often effective in BRCA1-associated cancers due to their defects in DNA repair mechanisms, was appropriate [12]. However, the recurrence of TNBC despite aggressive treatment further emphasises the need for continuous and intensive surveillance.

In terms of cervical cancer treatment, this patient underwent concomitant chemoradiation therapy, which is the standard of care for locally advanced cervical cancer. The presence of a BRCA1 mutation might also influence the decision to consider prophylactic surgeries or interventions in the future, such as oophorectomy (removal of the ovaries) to reduce the risk of ovarian cancer [13]. Such decisions require careful counseling about the risks and benefits of preventive measures, especially when the patient is already dealing with multiple cancers.

This case also raises broader questions about the need for heightened awareness of the risks associated with BRCA mutations. As genetic testing becomes more common in clinical practice, healthcare providers must be diligent in considering the implications of these mutations across various cancer types. For example, in patients with BRCA mutations, routine surveillance for cancers outside the breast and ovaries—such as cervical, pancreatic and colorectal cancers—should be considered. This personalised approach to surveillance and prevention could potentially catch second malignancies earlier, improving outcomes and survival rates.

Finally, this case emphasises the need for an integrated, multidisciplinary approach in the care of patients with hereditary cancer syndromes. The management of such patients often involves not just oncologists, but also genetic counselors, surgeons, radiologists and other specialists who collaborate to optimise care and surveillance. Tailoring treatment based on the genetic background of the patient and understanding the interplay between different malignancies in the context of BRCA mutations is crucial for improving both quality of life and survival in these complex cases.

## Conclusion

The co-occurrence of recurrent triple-negative breast cancer and cervical cancer in a patient with a BRCA1 mutation underscores the importance of genetic factors in the development of multiple primary malignancies. It highlights the need for personalised treatment plans, vigilant surveillance and genetic counseling to optimise outcomes for patients with hereditary cancer syndromes. This case serves as a reminder of the multifaceted risks associated with BRCA mutations and the importance of a holistic, individualised approach to cancer management.

## List of abbreviations

BIRADS, Breast Imaging Reporting and Data System; BRCA, Breast cancer; PARP, Poly (Adenosine diphosohate-ribose) polymerase; DNA, Desoxy-ribo-nucleic acid; FDG PET, Fluorodésoxyglucose positron emission tomography; FIGO, Fédération internationale de gynécologie et d’obstétrique; HER2, Human Epidermal Growth Factor Receptor-2; HPV, Human papillomavirus; MRI, Magnetic resonance imaging; SBR, Scarff, Bloom and Richardson; SUV, Standardised uptake value.

## Conflicts of interest

The authors declare no conflicts of interest related to this work.

## Funding

This publication was prepared without any external source of funding.

## Informed consent

There was full compliance with ethical standards including informed consent. The patient has provided verbal consent for the publication of this report and any accompanying images.

## Authors contributions

Naciri Meryem: Conceived the clinical vignette and wrote the manuscript.

Aouzah Fatima Ezzahra: Collected and analysed the data and contributed to the writing of the manuscript.

Kouhen Fadila, El Ghanmi Adil, Ghazi Bouchra, Sqalli Houssaini Mohammed, Fichtali Karima: Reviewed and edited the manuscript and provided critical feedback.

All authors have read and approved the final version of the manuscript.
